# Art-Science Collaboration in an EPSRC/BBSRC-Funded Synthetic Biology UK Research Centre

**DOI:** 10.1007/s11569-020-00367-3

**Published:** 2020-04-21

**Authors:** Michael Reinsborough

**Affiliations:** 1 University of West of England, Bristol, Frenchay Campus, Coldharbour Lane, Bristol, BS16 1QY UK; 2 BrisSynBio, a BBSRC/EPSRC Synthetic Biology Research Centre, Life Sciences Building, Tyndall Avenue, Bristol, BS8 1TQ UK

**Keywords:** Responsible Research and Innovation, Synthetic biology, Art-science collaboration, Immersive theatre, Public engagement, Interdisciplinarity, Cross-sectoral exchange

## Abstract

Here I examine the potential for art-science collaborations to be the basis for deliberative discussions on research agendas and direction. Responsible Research and Innovation (RRI) has become a science policy goal in synthetic biology and several other high-profile areas of scientific research. While art-science collaborations offer the potential to engage both publics and scientists and thus possess the potential to facilitate the desired “mutual responsiveness” (René von Schomberg) between researchers, institutional actors, publics and various stakeholders, there are potential challenges in effectively implementing collaborations as well as dangers in potentially instrumentalizing artistic work for science policy or innovation agendas when power differentials in collaborations remain unacknowledged. Art-science collaborations can be thought of as processes of exchange which require acknowledgement of and attention to artistic agendas (how can science be a conceptual and material resource for new aesthetics work) as well as identification of and attention to aesthetic dimensions of scientific research (how are aesthetics and affective framings a part of a specific epistemological resource for scientific research). I suggest the advantage of specifically identifying public engagement/science communication as a distinct aspect of such projects so that aesthetic, scientific or social science/philosophical research agendas are not subsumed to the assumption that the primary or only value of art-science collaborations is as a form of public engagement or science communication to mediate biological research community public relations. Likewise, there may be potential benefits of acknowledging an art-science-RRI triangle as stepping stone to a more reflexive research agenda within the STS/science communication/science policy community. Using BrisSynBio, an EPSRC/BBSRC-funded research centre in synthetic biology, I will discuss the framing for art-science collaborations and practical implementation and make remarks on what happened there. The empirical evidence reviewed here supports the model I propose but additionally, points to the need to broaden the conception of and possible purposes, or motivations for art, for example, in the case of cross-sectoral collaboration with community engaged art.

## Background: RRI and Synthetic Biology

BrisSynBio is one of six UK EPSRC/BBSRC-funded research centres in synthetic biology.^1^ Using tools such as CRISPR/Cas9 [1, 2], BrisSynBio researchers work to develop synthetic biology to do various things, for example to modify human red blood cells, cellular mitochondria or the DNA recombination machinery (for meiosis) of crop plants. Framed within a responsible research and innovation (RRI) agenda, BrisSynBio research must also ask to what extent the laboratory research agenda and/or laboratory practices are shaped by ethical concerns, public engagement or policy debates surrounding the future use of gene editing tools in medicine, fundamental research and food crops such as wheat. One way in which BrisSynBio has engaged with RRI is by deliberately using an arts theme. In this article, I will very briefly introduce RRI, synthetic biology and art-science collaboration so that I can describe how art-science projects might engage with science policy goals like research responsibility and provide suggestions for practice based on analysed empirical material from interviews and ethnography during my time as a participant at the centre.

### Responsible Research and Innovation

**Fig. 1:**
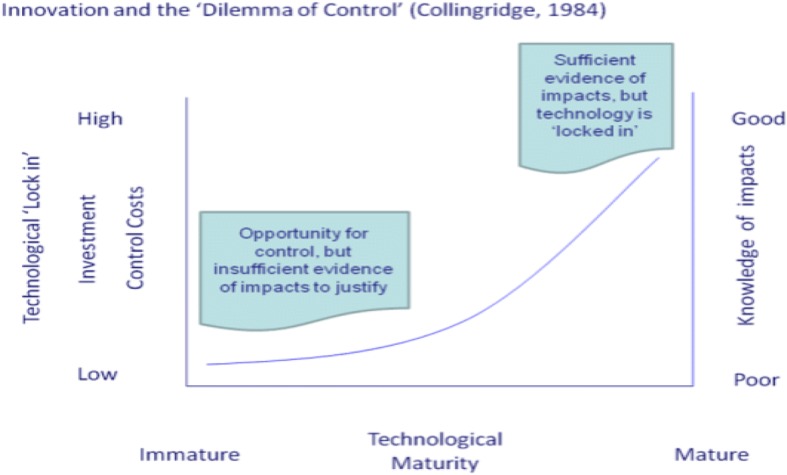
Collingridge Dilemma [8]

“Responsible Research and Innovation” (RRI) is a more recent policy term to describe a changing approach to the relationship between science and society in a changing research economy. In the previous linear model, the corporate research laboratory had the expected role of translating university research into something economically useful. In the post-Fordist research economy, the corporate research lab (a famous example could be Bell Laboratories) has largely disappeared [3]. Universities are expected to take up this slack having a more proactive role in regional economies [3]. Recent policy emphasis on competitive innovation economies has been paired with calls for RRI. The previous moral contract between scientists and society could have been summarized as “pay for basic science and leave scientists alone and this will, in the long run, generate applications of benefit to the economy and society”. But this previous rhetoric regime justifying funding, decision-making and research direction is now largely being replaced. A new “Grand Societal Challenges” model suggests research should be useful to society and address grand challenges in society and/or global society. One reason for this has been a long-term recognition of the Collingridge Dilemma: that with new technologies, we have not enough knowledge about their environmental, health, social and safety impacts to effectively regulate them; but by the time we do have enough knowledge to do so, the technologies are well established (path-dependency) and therefore difficult and expensive to change [4].^2^ Lastly, there is a growing recognition that what begins in the lab (if it is successful) becomes a generalized product, technique or knowledge outside the lab. An awareness of how the research process itself is the beginning of any outcomes and that choices in the laboratory may have longer term consequences (path-dependency) that are difficult and expensive to correct at a later date when research-generated industrial processes are in place and the innovation has become embedded in existing social and technological systems. At that point, it becomes very costly to make changes, even if we can now understand and justify what could be made better, or at the very least less harmful (Fig. 1).

Therefore, a question which RRI attempts to address is how we might pull society and its concerns and needs into the lab earlier so that what comes out of the lab is already better integrated to the needs of communities. That RRI emphasizes not just “responsible innovation” but also “responsible research” indicates the break with the old linear model where scientific research was considered independent of its consequences, which were thought to only accrue later in an applications development phase.

An important early policy framing for Responsible Research and Innovation defines it as an “interactive process by which societal actors and innovators become mutually responsive to each other with a view to the (ethical) acceptability, sustainability and societal desirability of the innovation process” [9].

There are several models for integrating social/societal concerns into research agendas. For example, the EU model has delineated a list of policy areas to address (public engagement, open access, gender, ethics, science education) for institutional change.^3^ I will here focus on the so-called AREA model, promoted in the UK by the Environmental and Physical Sciences Research Council (EPSRC), successful at explaining the concept because it seems to encapsulate the complexity in a memorable way.^4^ AREA stands for Anticipate, Reflect, Engage and Act. The qualities of being anticipatory (being aware of how expectations of the future influence the present; using a multiple plausible scenarios approach), reflexive (researcher awareness of their own position within the greater research economy) and inclusive (broadly inclusive as a deliberative process, i.e. mutually responsive to dialogue with others both within and outside of the immediate research community) are to be integrated into research agenda (to act institutionally). While this does not guarantee consensus from all the various parties, it is hoped that one outcome will be changed research trajectories which begin to better reflect societal concerns.

I have here simplified the history and explanation of the term RRI which is undoubtably interconnected with several other similar concepts and has been taken up by different communities with different goals for slightly different reasons. The commercial recognition that design of technology matters in how potential customers take up products and the need for companies to be trusted by their customers has made Responsible Innovation a recognizable term in the commercial sector. An existing and evolving discourse within the social sciences, humanities and science policy has included ELSI/ELSA/various types of Technology Assessment (Constructive Technology Assessment, Parliamentary Technology Assessment, Real Time Technology Assessment etc.) which have all contributed to our academic understanding of RRI.^5^ Although it is not often acknowledged, the greater willingness to consider RRI might also be part of a desire to avoid previous political disputes during or after the introduction of new technology (i.e. social change actors who oppose the impact of particular technologies on their community, even calling for the democratization of technology design processes or scientific research trajectories). Previous controversy around GM crops is one example. There are many claims on what “responsible” might mean. The word responsible is a quite generic term, thus allowing different persons or institutions to take up RRI in different ways. In some ways, “RRI” is a fraught term—quite politically neutral in a realm where there are potentially conflicting commercial and civil society interests in how technological innovation establishes the framework for what is and is not possible in society, how decisions are made and how resources are distributed, i.e. the basis for politics in a technological society is the technical infrastructure of society [22]. It is also worth considering whether, or not, there are implicit claims in the word “innovation” which is part of the term “RRI”. Recent policy emphasis on competitive innovation economies within a neo-liberal economic growth agenda has been paired with calls for RRI. In contrast, some academics have asked, why not choose “Responsible Research and Stagnation” in a world where innovation-driven growth puts the economy on a collision course with the planetary boundaries [23].

The practical application of RRI requires knowledge production to be *anticipatory*, *inclusive*, *reflexive* and *mutually responsive* [9, 21] as well as *integrative* [21] Aicardi et al. [24] of all of these aspects within a research and ethics work programme. To be inclusive is also to develop relations with stakeholders and publics outside of the academy and thus requires more than just *interdisciplinarity* between researchers; it needs the development of *cross-sectoral* sensibilities. Like interdisciplinarity, cross-sectoral exchange also requires attention to the differing interests and common practices of communities with different purposes, but if anything, the challenges of cross-sectoral exchange are more diverse.

How do we integrate these different types of knowledge (research, engineering and community)? A first step in this process is interdisciplinary cooperation between expert academic fields. Cooperation between different epistemic cultures [25] presents a number of challenges. For different disciplines, what constitutes a valid research question and the type of evidence required to answer that question may vary. Because of this, how resources are allocated and what individual researchers must do to progress in their career may be different. Even scientific terminology that sounds the same (homonymic) may be applied very differently between disciplines, having in practice effectively different operational definitions for the same term. Interdisciplinarity may require additional resources and time to bridge these gaps. With these greater differences, collaborative projects often require greater time for trust to develop between parties. Short-term projects may fail because of this.

But the task of “putting society in the lab” suggests thinking a little bit further than just the challenges of interdisciplinarity. Interdisciplinarity is cooperation between academic disciplines within the academic sector. Cross-sectoral cooperation is cooperation between different sectors (i.e. between the academic sector and the private commercial sector, or civil society, or the government/policy sector etc.). Attention to cross-sectoral differences matters because just as there are different incentive structures and interests in what counts as knowledge between disciplines (different epistemic cultures) which complicate cooperation, so also there are entirely different goals, or even values, between sectors. While a great deal has been made of interdisciplinarity in research, less attention has been dedicated to the cross-sectoral requirements of research that wants to be responsive to societal concerns, desires and needs.

I want to turn in the next section to one proposed aspect of cooperation which lies somewhere between the interdisciplinary and the cross sectoral, that of art-science collaborations and what role they might have in an RRI programme for a synthetic biology research centre. But first, it is worth noting what synthetic biology is, and some of the connotative similarities which it might have with performance in art, especially when synthetic biology is considered as an action where its own definition is at stake.

### New Biology

Molecular biology includes laboratory practices to isolate and work with cellular DNA, the genetic material that directs protein construction in the cell, and thus in part directs the development of living matter in the cell or the larger organism. Bioinformatics is the use of computer analysis to identify patterns and useful information with DNA information abstracted from the cell or the organism by molecular biology techniques such as PCR [26]. The idea that DNA can be extracted, analysed, understood, manipulated and reinserted into the organism to create different organizations of protein and therefore different forms of life is key to the concept of synthetic biology. Thought of as a purely epistemological practice synthetic biology is the manipulation or even synthetic reformulation of life^6^ so as to understand how biology works. However, synthetic biology is most often thought of as an engineering practice for biotechnology applications by manipulating the metabolic pathway of a one-celled microorganism to generate a useful output, a cell that takes in a feedstock and outputs an organic molecule of some industrial, pharmaceutical or food engineering use. Applications to multicellular organisms are also being considered in some of the research at BrisSynBio and elsewhere.

A group of early starters in the field, molecular biologists/bioinformaticians at a few important institutions, have demonstrated in the laboratory proof-of-principle evidence of the synthetic biology concept and promoted the idea more broadly, including to science policy actors, funders and commercial investment actors with promises of future application [27]. As a concept, synthetic biology invokes several interdisciplinarities (biology, engineering, chemistry, computing) but also stands at the cross-sectoral boundary between the university and entrepreneurial biotechnology enterprise. The question remains whether the concept translates well to ordinary biologists further from this “core” of synthetic biology researchers, institutional resources and know-how.

The attempt to formulate synthetic biology as an academic research discipline or subdiscipline with a role identity for those who practice synthetic biology is an important aspect of resourcing such work at the changing intersection of university research economies. Synthetic biology researchers would then have departments and positions to take forward their career, academic journals to publish in and, as a recognized discipline, funding to initiate new research.

The performance of synthetic biology (as a type of role identity) is fraught. Balmer et al. [28] note some of the role identity difficulties of successfully performing oneself as a synthetic biologist and particularly the differences in this task for those in the core and at the periphery of a field of social action to establish such a proto-discipline. With less resources and less ability to define what counts as synthetic biology, new researchers at the periphery of the discipline find it much harder [28]. This is particularly true of PhD students taking up the field as the hopeful beginning of their career in molecular biology.

While the ideas of synthetic biology might be available for researchers, they must be enacted. The biologist must claim resources for a synthetic biology research project, and enact the claimed relations between a microorganism, the manipulation of its metabolic pathway and a useful outcome. Then, translation of tentative laboratory results to a successful industrial process within a viable business model and appropriate investment capital can be even more difficult. Thus, the successful enactment of being a synthetic biologist exists at multiple levels of performance: as role identity, firstly as academic, attempting to formulate synthetic biology as a discipline or subdiscipline; and secondly as entrepreneur, in the parallel attempt to enact the ambitious claims of early synthetic biologists within biotechnology innovation economies (usually imagined as start-up companies driven by a scientist-entrepreneur hybrid); and thirdly, a performance within microorganism metabolic economies (a somewhat more ontological performance—can living processes be made to be engineering processes?).

The difficulties of this have made synthetic biology a challenging and potentially changeable proto-discipline to enact. The original expectation of synthetic biology as an almost unlimited magic factory for anything largely failed to account for the costs of molecular metabolism. Cellular production, even if one can successfully manipulate DNA, tame the promiscuous and changeable character of microorganisms to create a stable laboratory strain, and translate that to a manageable industrial process (no mean feat), is itself costly in several ways. It turns out that molecular metabolism is most successfully performed with high energy feedstocks such as sugars which are commercially expensive, in many cases more so than the proposed output. In practice, this has meant that many early career would-be synthetic biologists confront the threat of failure, particularly if they depart from the academic career path to pursue an exciting but risky entrepreneurial (or hybrid entrepreneur-academic^7^) role. The previously open-ended economic narrative of the benefits of synthetic biology is now more likely to settle on a few plausible high value organic molecules such as flavourings or food additives (vanilla, menthol or stevia are good examples). Applications in biomedicine or antibiotics research are still active concerns. One BrisSynBio start-up company is developing underwater industrial adhesive applications and has entered into research contracts with the British navy. While synthetic biology is by no means exhausted or ending, the initial pre-conceptions of the field may be adapting to experience; and as Balmer et al. [28] note, this is especially true in the relation between the “core” and the “periphery” of synthetic biology.

The concept of “performing” synthetic biology has an interesting resonance with performance in art, which I will mention only briefly without attempting to draw too close a parallel. That one’s work depends on the successful performance of that work provides a parallel between the artist on the one hand and the early career molecular biologist/aspiring synthetic biologist on the other. The word performance has a wide variance of connotations. A performance in theatre is not quite the same as a performance of music. No one would say of a musician that they were not a musician because what they were doing was only performing music, whereas an actor performing Hamlet would be recognized as pretending to be so. The performance of a social role is somewhere in between pretence and enactment, at least initially, as the social agent sets out to undertake a role for which they do not initially have the necessary credentials and recognition. The phrase “fake it ‘til you make it” is a common sense understanding of this.

In the social sciences, symbolic interactionism and ethnomethodology have traced the path between a social theory of pretence (for symbolic interactionism there is an “off stage”) and one of enactment. Actor-Networks Theory, a later development of ethnomethodology [29–31], even takes up the role of materiality in enactment. Human enactment of a role is complicated by and imbricated in the materiality of the practice, for example, the molecular biology equipment, computers and software for bioinformatics, and not least, the uncertain role of microorganisms who cannot always be “performed” exactly as hoped.

Drawing a parallel to dance, Myers [32] has suggested that molecular biology is also a bodily performance. Indeed, in my fieldwork, I observed a senior molecular biologist instructing others and enquiring about their method of placing a tip on their pipette (tool for administering very specific amounts of a fluid; the key activity of molecular biology is mixing with pipette different carefully measured amounts of fluid, genetic fluid, reagents etc.). To tap down one’s next disposable pipette tip was a routine activity to make sure it was properly attached but how one did this could vary. Would they use one tap or two? Would they have a specific rhythm or cadence? What was being suggested was that each molecular biologist could do so in a specific way, repeatedly, that is to have a signature tap, specifically (and self-consciously) chosen by themselves. This was encouraging uptake of performance and a clear role identity with the body and its habitual gestures self-consciously invoked in the performance of molecular biology. While quite obviously performance is enactment (imagine the sceptic saying, “molecular biology involves tasks with equipment, chemicals, reagents, material, and yes we do this with our body, so what?”), consider also that there are many different ways that the body could do this, and by drawing attention to this bodily performance (new biologist: “I choose more precisely how I will perform or enact my laboratory activity”), you may become a better molecular biologist. And thus the embodied action of molecular biology in this instance notes playfully a small nuance of performance or flourish somewhere between pretence and enactment. I speculate it may have benefit in the overall quality of performance of molecular biology when the laboratory experimenter develops specificity (and pride) in their manual method. A more thoughtful pipette user (even if that thought is simply at the level of bodily action, i.e. tacit knowledge) is a better pipette user, i.e. more likely to be a successful molecular biologist. As I could wirness, many tasks in molecular biology are very difficult to perform, such as isolating genetic material using chemical reagents, the pipette and the human hands.

With this brief interlude (and again I do not want to make too much of a relatively small moment of flourishing within a discipline that is very much about tacit knowledge), I will pass on to discussing art-science. But the very small point I want to draw out of this incident is that bodily performance is an aspect of science which although present, is rarely considered greatly by laboratory scientists. Who might more likely if asked define science as an epistemological enterprise, neglecting its daily routines and more mundane activities, however skilled and precise they must be. Bodily performance and role experimentation is also something explored in the arts, for example theatre.

## Art-Science Exchange

While art-science collaborations offer the potential to engage both publics and scientists and thus possess the potential to facilitate the desired “mutual responsiveness” (René von Schomberg) between researchers, institutional actors, publics and various stakeholders, there are potential challenges in effectively implementing collaborations as well as dangers in potentially instrumentalizing artistic work for science policy or innovation agendas.

### Art-Science

Let us begin with a simple theoretical framework to guide how we might think about an art-science collaboration: Art-science collaborations are processes of exchange which require acknowledgement of and attention to artistic agendas (how can science be a conceptual and material resource for new aesthetics work) as well as identification of and attention to aesthetic dimensions of scientific research (how are aesthetics and affective framings a part of and a specific epistemological resource for scientific research). The value of this two-way exchange process can be initially difficult for either party to perceive because of disciplinary pre-conceptions that members of either group may have, either specific to their group or shared. The very function of science as a successful disciplinary practice emphasizes disciplinary fidelity. That biologists adhere to collectively understood rules of what constitute legitimate biological questions of interest and valid rules of evidence collection or experimental method to answer such questions, is the basis of such disciplinary framework. Because they remain within it, they retain access to disciplinary resources such as publication opportunities in biology journals, research funding and career progression opportunities, which become available to them exactly because their work fits the criteria for the biological sciences. Similarly, while the arts are perhaps a broader set of fields than molecular biology, there are here also given practices, ways of looking at the world, questions that seem of interest that are legitimate to ask as part of artistic research and questions that seem outside of the artistic remit.

Generally, scientists (molecular biologists included) do not understand themselves to be doing work in aesthetics, their emphasis instead being on determining natural laws empirically through hypothesis formation, experiment and empirical verification/falsification. Science is seen to be an epistemological project about the nature of the world whereas aesthetics, as defined by seventeenth century philosopher Alexander Gottlieb Baumgarten, is a practice to organize or relate our sensory experience to a cognitive process [33].

**Fig. 2:**
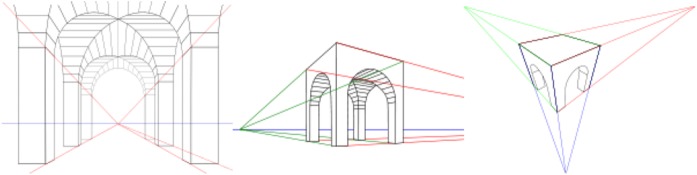
One-point, two-point and three-point perspective. Image from https://en.wikipedia.org/wiki/3D_projection

Thus, a simple example one might use rhetorically to explain aesthetics to a molecular biologist might be the role of perspective in Italian renaissance painting. The sensory experience of seeing perspective (depth perception) was translated in a formal way to two-dimensional representation by creating specific rules for perspective [34–36] (Fig. 2).

While today we might feel representation on a two-dimensional surface of our visual experience of seeing in three dimensions (how our human brain represents depth perception to ourselves) seems quite natural this is in fact a learned way of seeing. For thousands of years, representation on two-dimensional surfaces had been without the formal rules (scientific) of a topology. This new way of seeing pictures was initially a matter of artistic innovation—a new way of relating the sensory experience (of a strangely marked two-dimensional surface) to our cognition.

**Fig. 3:**
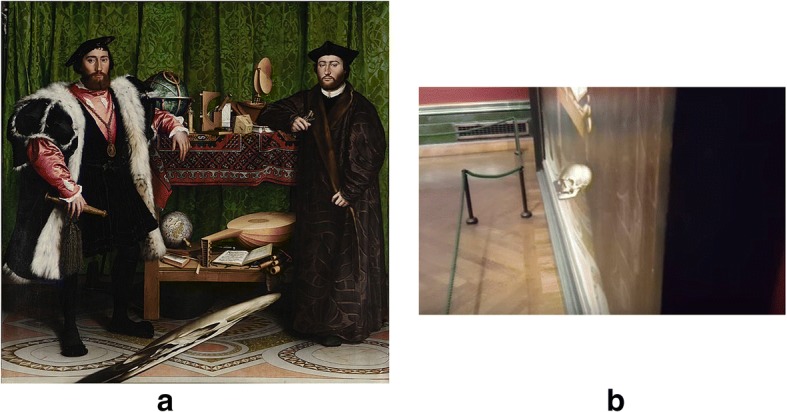
**a** Holbein’s “The Ambassadors” with anamorphic projection (skull). **b** Lower portion of the painting viewed from an angle. Images from https://en.wikipedia.org/wiki/The_Ambassadors_(Holbein) and https://www.moillusions.com/the-ambassadors-a-3d-painting-by-hans-holbein/

In the picture “The Ambassadors” (1533), the artist, Holbein, demonstrates humorously that he and other artists were well-aware of the slightly arbitrary nature of these rules by showing a topologically represented skull, blurred by normal rules of representing three-dimensional perspective on a two-dimensional surface but available to a viewer of the portrait who sees it from an angle. These slightly different rules of topology are called anamorphosis [37] (Fig. 3).

This definition of aesthetics, although commonly hidden by disciplinary prejudice, is clearly present in the practice of science. Sensory perception must be organized to fit a cognitive procedure or choice in a variety of scientific experiments. Early biology with microscopes required the development of staining processes that allowed a cell to be seen and drawn. Certain aspects of the cell, like the cell wall, became visible in this process. This certainly seems like a way of organizing sensory experience to a particular cognition or understanding. Likewise, today, a huge part of scientific papers published in peer-reviewed journals is the organization of empirical evidence visually in figures, for example, a colourful graph or chart, or selecting/preparing a data photograph. In some cases, some choices must be made; convention will not cover all circumstances; and these choices are in part aesthetic, choices about how to organize sensory experience in a way to make it fit to a cognition. How a landscape painter makes sensory experience clear to an audience and how a scientific paper does so are very different, with very different goals, practices and conventions, but both are to a lesser or greater extent, and with different emphasis, and not as their only purpose, involved in some forms of activity that fit Baumgarten’s definition of aesthetics: aesthetics as a practice to organize or relate sensory experience to a cognitive process.

Reciprocally, the renaissance example of developing a perspective aesthetics demonstrates how epistemological processes aimed at understanding nature (in this case, the science of topology) can also be a resource for developing new artistic practice.

So with this basic demonstration of how aesthetic and epistemological practices overlap, I repeat the theoretical framework by which we might understand and potentially guide art-science collaboration. This definition can be helpful in setting up, assessing or guiding art-science collaborations. Art-science collaborations are processes of exchange which require acknowledgement of and attention to artistic agendas (how can science be a conceptual and material resource for new aesthetics work) as well as identification of and attention to aesthetic dimensions of scientific research (how are aesthetics and affective framings a part of and a specific epistemological resource for scientific research). They are an exchange, an exchange which can only be successful if neither art nor science is fully objectified by the other discipline.

When power differentials in collaborations remain unacknowledged, it is often the scientists (with more economic and social capital leverage than artists usually) who are more likely to objectify or instrumentalize art for their own agenda. A common objectification of artistic work within an art-science collaboration is to see it as a resource only for public engagement, and at worst a propaganda tool for the laboratory researchers’ desire for “public understanding of science” grounded in a deficit model of the public, that the public lack (have a deficit of) scientific understanding and that that is the only reason for some critique or disagreement the public might have with a scientific project. There are institutional desires to be celebratory about the work that researchers do and about the value of science, without critical engagement with the ways in which scientific research is imbricated in the social, political, economic, environmental and cultural structures of society.

Occasionally scientists will fear that the artist/writer/painter will take up negative images of science influenced futures, that the attempt to interest an audience will cater to the dystopian. Will the anticipatory aspect of imagining futures (derivative of or influenced by new science) be plausible? I have elsewhere written of plausibility in science fiction, or so-called near-future fictions [38].

I suggest the advantage of specifically identifying public engagement/science communication as a distinct aspect of such projects so that aesthetic, scientific or social science/philosophical research agendas (if present) are not subsumed to the assumption that the primary or only value of art-science collaborations is as a form of public engagement or science communication to mediate biological research community public messaging.

But the failings of scientists are not the only concern. Artists also can fail to take seriously the science. James Elkins argues that artists must work to understand what it is that science is trying to do on its own terms, rather than simply take up imagery or some other product of scientific craft as an attempt to accomplish that for which an artist might use such material [39]? Taking science seriously does not mean being uncritical or taking science too seriously. In addition to outright misunderstandings of scientific claims, artists might also take up sensationalist portrayals of research results or uncritically promote scientific visions, fictional futures or other ideas about the relevance of scientific research to parts of the world outside the promoters remit or expertise. Another common failure is art based on a superficial examination of science’s final outcome—rather than examining fully the method and materiality that combine to become the epistemological claims [40, 41]. Borrowing from sociological studies of science and technology, Silvia Casini suggests a method for artists to “open up the black box”^8^ of science if they desire to critically engage [41]. Art that helps us rethink the role of science in society (or even the role of science in art) examines the instruments, material and craft-based knowledge construction of the scientific field that it seeks to explore, comment on or utilize as a resource for new art practice. Another similar failing is to not see art as interpenetrated with the craft and culture of other spheres of the world, most notably for the purposes of this essay, science. That art can have an experimental method and that science might contribute to rethinking artistic processes and experiment (particularly when exploring new materials, instruments or ways of mixing realms of world that new science makes possible) are important points that a successful art-science collaboration might bring out for artists.

In a successful exchange, each party takes the exchange seriously. Either party may see the benefit of the collaboration as something different than what the other sees as the benefit. Different aspects of collaboration or its outcomes may be seen as useful to different parties. Problematically, conceiving of art-science as an exchange can create expectations that more will be accomplished than might actually be achieved. When setting expectations, it is important to realize that while aspects of science contain aesthetic practice, which might be useful for scientists to have a reflexive awareness of, and aspects of art practice have roots in science mediated epistemologies of the world, neither art nor science are primarily improved, or succeed within their own disciplines, through or because of these modes. A collaboration may contribute something to each discipline, but it may not contribute that much.^9^

So I suggest two simple rules to guide an art-science collaboration: that there is some benefit to each party, however small, and even if that is not necessarily a common benefit; and that each take the other seriously.

### Art-Science-RRI

So with this simple framework and set of rules, let us consider art-science collaborations in relation to implementing a programme of Responsible Research and Innovation as science policy goal. Is there a potential for art-science collaborations to be the basis for deliberative discussions on research agenda and direction.

How could art-science collaborations accomplish this? Using the simple AREA model (Anticipate; Reflect; Engage; Act), it is easy that to see art-science projects can develop anticipatory frameworks that allow scientists and publics to explore how they feel about different potential future scenarios. Causing publics/scientists to see their own location within one or another of the futures, and thus also consider their own location relative to creating/preventing potential futures encourages reflexivity. Immersive theatre is an example used at BrisSynBio and discussed below. The ability of art to present material in accessible and interesting formats allows complex topics to be presented to a greater variety of people, many of whom would simply “leave to the experts” discussions of science in traditional format.

Weaving together an anticipatory, reflexive and inclusive discussion may be a possibility for process-based art projects emphasizing participation—but ultimately integrating the outcome of such a process into the research agenda is then the responsibility of the research programme itself.

I began by describing a theoretical framework for art-science collaborations as an exchange, an exchange which can only be successful if neither art nor science is fully objectified by the other discipline. Therefore, although I do not discuss this point at length, it goes almost without saying that an art-science project should not, in a similar manner, be instrumentalized or thought of instrumentally for the achievement of RRI science policy objectives. The social scientist, the philosopher, the embedded humanist, the science policy agent, the research officer, the funder, the scientific researcher, whoever it is that facilitates or promotes genuinely the RRI strand of scientific research, must also take seriously the aesthetic and epistemological goals of an art-science project. They must consider an art-science-RRI triangle. Art-science-RRI collaborations must consider the interests of all parties in the exchange and take seriously in equal measure the epistemological, aesthetic and reflexive purchase of such an exchange.

## Empirical Results

The Responsible Research and Innovation component of BrisSynBio was to be organized as a cross-cutting thematic strand of the overall project. This strand was overviewed by a Principle Investigator (social sciences) and lead by a Director of RRI (philosophy) both of whom came from the University of the West of England, Bristol, while the overwhelming majority of the synthetic biology project teams were from the life sciences and chemistry at the University of Bristol. When I joined (two years into a five-year project), there were already a number of activities in place for the RRI theme. The week I arrived, an exhibition of a half dozen artists commissioned to do work on a synthetic biology theme held an opening in a pop-up gallery in the Old Market neighbourhood of Bristol. Researchers from the synthetic biology centre came to see it. Two “ethics of” case studies were already in place (synthetic or “cultured” blood; and life/non-life boundary at the molecular level [43, 44]. And another was to be started (wheat) by myself. Various events organized by the RRI theme had already happened such as a synthetic biology in society blog and a large open public discussion on synthetic biology.^10^ Synthetic biology PhD students within their training programme (South West UK cohort) had time dedicated to ethics and society topics, which the BrisSynBio RRI team helped deliver. There were also social sciences and philosophy PhD students undertaking research on society, philosophy and ethics issues at BrisSynBio. The second BrisSynBio annual conference, unlike the first, would include a keynote RRI speaker (myself). An RRI theatre event (or as I would later learn, a “theatre process” culminating in a theatre event) had already happened and, as I noted with interest during my week of arrival, people were very pleased with it (discussed below). A philosophy of synthetic biology conference was planned to happen (of which this special issue is an outcome). In the works was an away day weekend in Wales for early career researchers and PhD students focused on RRI and art. The theme of Responsible Research and Innovation was just beginning to flourish in the research centre. Over the course of the next two years, more events would be organized including a conference bringing together social science and philosophy researchers in the UK working on the topic of RRI in synthetic biology and contributions to national and international science governance consultations.

I point to the specific way that an interest in art seems to have become a useful format with which biologists at the centre could understand and try to fulfil their RRI obligations. The centre had chosen to organize RRI work along a specific strategy and this emphasis on linking art to science had emerged.

Why? Some scientists seemed to like it. And it created a focus for RRI responsibilities which might otherwise have seemed ethereal to the biologists. An early project in theatre funded as part of the EU project Synenergene had worked with the research centre. The positive experience which came out of that early project seems to have encouraged this direction.

For the sake of brevity, I will not be able to document all of the work but in line with the question of how art-science might be used as part of an RRI program, I will discuss just a few examples, the initial theatre project, some of the framing for art-science at the centre and PhD student experience in relation to art-science and RRI. My fieldwork data was generated over two years of ethnography and includes two dozen interviews, three of which I incorporate here. Where useful, I also draw in ethnographic data from my ongoing participation in the cereal genomics lab, in various research centre events and talks, and from participation on BrisSynBio committees such as the Public Engagement Committee. Importantly, I have also been involved in other art-science projects (both successful and unsuccessful projects—sometimes one learns more from what’s missing from projects that do not succeed than what’s present in those that do) and this provides me perspective with which to better assess the BrisSynBio engagement. Although my background experience and this larger body of empirical material informs the theoretical framework I provide, I have chosen to illustrate this case by close reporting of a small selection of interviews. In this way, I hope to provide the reader a sampling of the detail of such a large project as perceived from different perspectives (what might be described as a “close reading” of the material) at the same time as providing the reader an overall framework by which to judge the empirical material for themselves. In the way I have presented it, it can loosely be seen as “before”, “during” and “after” a seminal event, but it is only a sampling of overall RRI activity at the centre, only some of which I was able to collect data on, and only some of that data was relevant to art-science collaboration.^11^

### Interview: Project Framing

The requirement that the molecular biologists engage in “responsible research and innovation” was an early mystery for them. A more senior scientist involved in the leadership team at BrisSynBio explained to me that they had worked on the synthetic biology components network, a project leading up to the funding application for BrisSynBio. This project had also had an ethics and society component. Of this my informant said, “We worked to understand it. How could we do this? Who were the key players?”

The RRI strategy of the centre was driven by those recruited to lead this theme, a sociologist and philosopher respectively. There were three specific case studies, and various centre-wide activities primarily focused around early career researchers. My interviewee explained to me that there were some early challenges in developing the ethics and society component of the programme. It took “a long time to find a common language between life scientists, philosophers and social scientists”. There was at times “resistance” to RRI from “some synthetic biologists and more so from senior or more established individuals”. Considering all the other challenges that synthetic biology as a science enterprise and role performance already presented, the additional work in a different and perhaps to some, seemingly off-topic direction, may not be what all senior researchers want to be doing. There was also the threat of academic overload, “…with everyone expected to do more and more and more and speak more and more different languages… we wanted to protect early career researchers from being pulled in too many directions at once”.

According to my interviewee, the creative role of art in the centre had not been strategic; it was never written down in a document. It had emerged in an “organic” way from the process of interacting with artists during the first two years of the Centre. For example, an artist worked with one of the case studies to explore blood culture and the aesthetics of blood in art, receiving multiple rounds of funding. The Bristol Centre for Public Engagement who had been involved in supporting BrisSynBio at an early stage brought several EU projects providing additional funding such as Synenergene^12^ and PERFORM^13^—looking at public engagement around synthetic biology, and exploring the arts as a tool in public engagement. The responsibility of the Bristol participants in the 20+ partner project Synenergene was to explore and conduct public engagement through theatre. This seems to have had a significant impact.

“…we obviously always thought that we’ll be able to count, y’know, the number of people that came, and the amount of schools that were involved, but what we absolutely hadn’t foreseen was that the process of writing this piece of theatre that we did our best to set up in such a way that it would draw on many people across BrisSynBio, we hadn’t foreseen that that process would be so empowering for the people that were part of it”.

The process of writing the piece of theatre was described as “empowering”, involving 18 months of interactive scriptwriting, working with a group of early career researchers. The directors and scriptwriter interacted with all BrisSynBio research groups and came back with questions. “There was time and permission to really think about these issues, to have that conversation”. This early success helped a group of scientists who had been wrestling with how they would do RRI at their centre come to a better understanding of what they might accomplish.

### Interview: Performing Artist

When I spoke with one of the directors from the theatre group which brought together the production, he discussed this careful interactive process. While his background in science was not strong, he clearly indicated that their first step in the process was to engage with the synthetic biology research at hand. One had to have curiosity and the ability to listen well and translate it back, he informed me.

They had been approached by the public engagement unit at the University of Bristol, had been given a few parameters, for example, the target age group for the audience was secondary school children, but largely there was little guidance given them. Interestingly, he saw the distinction between art and science as artificial, particularly when collaborating in an open interactive process. That both art track and science track school children were recruited as an audience he saw as positive, an early unravelling of the distinction. The play was considered very successful and recommissioned for a second run, this time aimed at a more general audience.

“What we bring to the table, what we are experts in is story-telling, having a narrative arc that will engage and include a whole lot of different perspectives and come across to an audience from a whole diverse range of backgrounds in an accessible and engaging way. Story-telling is character, understanding people. We look a lot at different futurological scenarios from working with science. We might try to imagine where it could be in twenty, thirty, fifty, a hundred years-time. But then, we think about the people that will inhabit those worlds, who fundamentally from a human psychic perspective might not fundamentally have changed. So to think about people from different parts of society, different ages and backgrounds might react to scientific development”.

Although he felt it was very important to have as much accuracy as they could feeding into the process, and thus working with scientists was the only way his theatre group would even consider doing such a piece, he was clear that they were not pretending to be predicting the future. The role of thinking about the future was less about emphasizing a plausible future scenario than creating a conversation about how humans interact with one another and why this might be relevant for doing science now in a way that might better acknowledge those interactions. The focus was on building a process rather than a product. The emphasis was on the journey and the legacy rather than the performances themselves. There were workshops and conversations with different groups in BrisSynBio. They wanted to build a collaborative process. There was a first draft public reading of the play to get feedback. The rehearsal draft was open, and people could come and comment. They asked questions and encouraged people to think in different ways. “Perhaps not to the degree that we were but the scientists were [also] co-authors of the piece”, he informed me of the play.

There were some challenges. Initially some had thought of how the arts could be used as a mouthpiece for science. Other researchers could not be involved because of limited time. Perhaps others thought it would not accomplish much or were not initially sure if it could. But this efficacy later became apparent in the process of building interaction and doing theatre exercises. “As much as possible we wanted them to see the potential of theatre, we invited them into our world, not just to talk about it but also to experience performance”. The molecular biologists were given to experience themselves as performers.

**Fig. 4:**
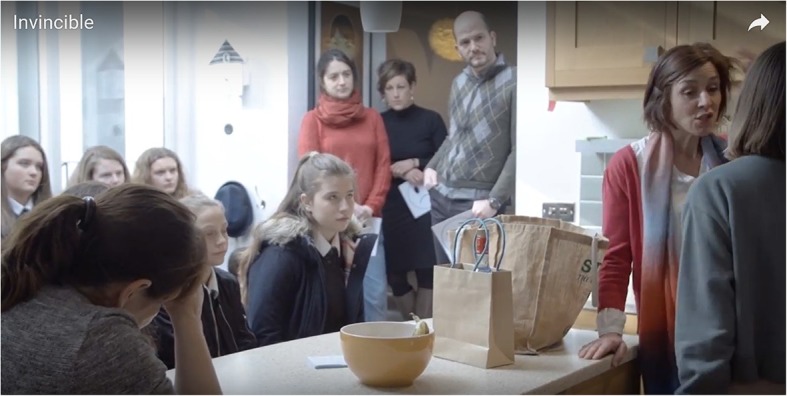
Immersive theatre: the audience in the kitchen of the action. Image from https://www.youtube.com/watch?v=71K6h3wg1i8

The subject of the play, *Invincible*, was chosen deliberately to be controversial: gene editing within the human being to remove a potential genetic mental health problem. The characters were three generations of women (grandmother, mother, daughter) and the grandmother had edited the mother such that the daughter was now different, changed in such a way that a presumed mental health problem was deemed less likely to occur. The play had a vicarious atmosphere—set within a domestic apartment, the audience came into an ordinary house whose kitchen/living room was where all the action happened (Fig. 4). Interaction with the audience was deliberate; between each scene, the audience were asked questions and a “scientist” in a white coat came out with a camera to make a record of the vote. Of this the interviewee said they were working not just to be a mouthpiece. It was very difficult to articulate questions that did not lead the audience in anyway. My informant explained that they wanted to sit in the middle, on the fence. They sought out questions which could get a 50/50 response from the audience. In this way, the provocation could continue. At the end, there was a questions and answers session with representatives from BrisSynBio. The scientists also asked questions of the audience. There was a deliberate attempt to reverse roles and upset the usual one-way conversations between scientific experts and “lay-people”. In this way, it was hoped that everyone could be considered an expert in what future was desirable for society. A film of the production was made, creating a documentary of the process and leaving a legacy of the play *Invincible*.^14^

The event influenced BrisSynBio in their early period of wrestling with what was RRI and how to engage with it. But it also influenced the theatre group. This had been their first longer engagement with an academic research centre but since then they have been involved in other art-science collaborations and elaborated further the process which they initiated first with BrisSynBio.^15^

When asked if policy (via the role of social science in the project) got in the way, 16 my interviewees expressed that they did not feel inhibited or directed. They felt they had a clear idea from the start of the job what the ambitions of those who commissioned the project were, and this is why they took it. If anything, they would have preferred to widen the audience, to throw it more open. And indeed, when the very successful performance was recommissioned, the target audience was broader, not just school kids.

### Interview: Art-Science and the Early Career Researcher

PERFORM^17^ funded the RRI retreat and by that time the leaders were quite deliberate about the arts-strategy because of the influence of the learning from the theatre process.

I interviewed two BrisSynBio members who were in their second and fourth year respectively of their PhD. They had come to synthetic biology from chemistry, and from chemical engineering respectively. Both had received brief introductions to RRI before during their Centre for Doctoral Training (CDT) sessions.^18^ The earlier CDT cohort had received slightly more training, about a week during their coursework and with a variety of speakers from different perspectives. Both had volunteered for the RRI retreat. They wanted to think more about the RRI aspects of their work.

The retreat was held in a Victorian mansion very much off the beaten track in the Wye Valley in Wales, down idyllic countryside lanes and over cattle grids. It was seen by them as location suitable for reflection. One noted how quiet it was relative to the city. Over the course of three days, the facilitators brought 15 participants through a series of games, creative exercises and discussions. There wasn’t a specific curriculum in relation to RRI but instead it was discussed more generally in creative processes such as painting, role playing, making clay sculptures, a walk through nature collecting items to attach to a cardboard “nature crown”, singing or choosing from a table full of trinkets and then discussing the object in relation to themselves and why they were there. In one exercise, participants stood along a line based on how much they agreed or disagreed with a statement, for example to what extent science was “objective” or “subjective”. As a group, most felt that science had significant subjective elements. In another exercise, they wrote Japanese Renega poems collectively, each one beginning a line of poetry and passing their piece of paper left to be continued by the next person. A professional singer composed a song from topics in an RRI discussion where participants had made statements and then discussed, identifying “hotspots” in the discussion where there might be disagreement, or where a topic was considered important. Participants were taught to sing these lyrics in multipart harmony, and with rounds. At the end of the three days, a number of summing-up discussions happened and participants were asked what they would take away from the weekend, what they felt they had learned. My interviewees noted that the weekend was very relaxing, despite being very “intense” in a certain way. They expressed positive feelings about the event. By being outside of their normal laboratory environment with a lot of time to talk and reflect, they felt that they were able to consider the importance of the topic to themselves in a way not normally possible in the day to day (sometimes stressful) activities of a research career. While it was great to have values and big ideas, everyday laboratory life did not always allow these to be enacted so, as one interviewee said, he would be “looking for those opportunities” when they did become available, reflecting and not making “brash decisions”. He wasn’t sure this would change anything he did immediately, but he would be reflecting on the bigger issues whichever career choice he made, academic or industry.

Both students who I interviewed had slightly different feelings of being disillusioned with science—the more senior had looked more closely and rethought science as part of the earlier more substantial week of training in RRI. He had previously thought of science as the “truth” but now thought in a more complicated way about science. He noted that it had a history of being associated with revenue generation for capitalism since the industrial revolution. As an engineer, finding better solutions was what he felt was most important, and in some cases, technologies presented as solutions were suboptimal—technologies made for the sake of it (to generate income, he felt). He felt the retreat gave him an opportunity to think through his ideas and made him more confident about speaking about science. The other student experienced some disillusionment as a result of the retreat—this had made him feel “sad” when he went away—that at the beginning of his PhD he had had what he described as a “naive” belief that science would provide the solutions—and now he felt that more realistically, science could be useful in some ways but it was not always a solution for every problem. He now thought about what technology was, how it impacts upon society and to what extent one could predict this impact. He spoke of undergoing thought about the development of science and technology. One interviewee remembered what one of the artist facilitators had said that they had learned during the retreat, “research scientists are left with this responsibility, and there should be stuff [resources] in place to help them”.

Both students commented on the interest and value of working with these other modes of learning, that their curriculum since GCSE^19^ had been almost entirely science based and that this was unfortunate, they felt. This resonates with remarks by the theatre maker, who noted the inclusion of both art track and science track students enabled them to see that art could be an unusual starting point to discuss science or that science could be a subject for artful performance. The interviewees felt that the science and art distinction was somewhat artificial, that science was a creative process. One example was being able to write well. The activities of the retreat had had effects, “changing operating patterns of how our brains work, breaking down barriers, creating mental fluidity”. It was “very relevant to use art as a way of exploring science. Every scientist could benefit”. Although it does not necessarily need to be about exploring RRI, he added.

In this last statement, he makes a distinction between what I would call an art-science collaboration and an art-science-RRI collaboration. It is perhaps a measure of the success of the process of that although art-science was used as a method to talk about RRI, the process in its participants estimation was not limited to that.

## Discussion of Findings

One measure of RRI process efficacy is to what extent the research agenda changes as a result of such processes (i.e. “mutual responsiveness” or “action” as the final “A” in the AREA acronym). The RRI retreat group was a smaller self-selected group of scientists from the overall cohort. Their influence on the overall cohort or the long-term research agenda of the centre is unknown. The theatre project brought in a finite audience and the audience influence on the research agenda of the centre is unknown. Since the research direction is set well in advance through funding proposals with long-term deliverables, any changes in research agenda could only emerge gradually, measurable across a larger interval than the period of this empirical research. But the self-identified influence on some individuals within BrisSynBio seems clear. The ability of the art-science process to invoke conversations that were anticipatory, reflexive and inclusive within the researcher community seems also evident. The art-science theme had not been organized strategically (pre-organized), but instead emerged organically. Even if the artist facilitators could invoke meaningful integration of different conversations, it is the researcher community that is ultimately responsible for taking appropriate action as a result of the process (Table 1).

**Table 1:** Immersive theatre impact measured against AREA model descriptors

Type of impact	Impact within researcher community (process)	Impact outside researcher community (product)
(A) Anticipatory	Low*	High
(R) Reflexive	High	Medium
(E) Inclusive engagement	Medium/high	Quantity: low quality: high **
(A) Action	Not measurable	Not measurable

*These ratings are my subjective assessment based on comparison with what might be possible or what has been achieved in other contexts (without correction for resources/budget expended). For example, “anticipatory” impact of “low” within researcher community is assigned in comparison with a Delphi study, whereas a rating of “high” for outside research community is given in relation to the type of conversations secondary school students might normally have about potential future influence of biotechnology on society

**While I do not have budget figures for the performance, production time relative to number of performances seems low. In principle, quantity and quality do not need to be inversely proportional since once developed, a high-quality engagement event can be made portable and repeated

This research documents similar types of resistance to RRI that other research into RRI elsewhere has found: lack of time, lack of belief that it will be relevant, and resistance to interdisciplinarity. But institutionally BrisSynBio seems to have been committed to fulfilling their (mandated) obligations to RRI, although how to do so was initially unclear to them.

This seems to be where art-science, not originally a specific strategic choice, emerged as part of BrisSynBio’s approach. Possibly because this emerged organically rather than strategically there seems little evidence in this case that a science policy/RRI agenda was problematic within an art-science-RRI triangle. With a pre-planned use of art-science directly mapped onto RRI objectives (a very different scenario than described here), this concern might in principle still be something to which a self-reflexive RRI team would need to be attentive.

**Fig. 5:**
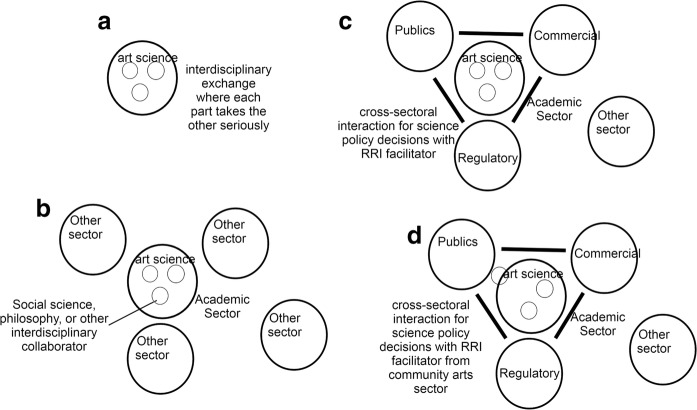
Interdisciplinary art-science collaboration (**a**, **b**) and interdisciplinary/cross-sectoral art-science RRI triangle (**c**, **d**). (In practice, the “cross-sectoral” boundary (which I emphasize here for conceptual clarity) is less distinct than I present because academic sector researchers have the world as a research topic and are in fact, like everyone else, in the world all the time. Cross-sectoral exchange, however, requires the aligning of differing goals, purposes and valuing practices (temporarily) between sectors and therefore is significant and identifiable within a sociological study.)

The reason I felt it was important to ask the artists about the impact of a science policy/RRI agenda upon themselves is explained thus. Art-science projects are modelled as interdisciplinary exchange which can be judged successful if each discipline takes the other seriously (Fig. 5a below). In my previous experience, these art-science projects had been set up or facilitated by myself and/or other social scientists (Fig. 5b).^20^ We had begun to think reflexively about our own role in the process. Therefore, we felt in an art-science social science collaboration we should be asking for feedback on whether we were thought to be taking our collaborators seriously. In addition to directly doing social science research, we were also agents of science policy emphasizing the responsible research agenda (Fig. 5c). In fact, the funding for the art-science projects came from this. Myself and my fellow social scientists had constructed an art-science RRI triangle where RRI was the facilitator. As social scientists funded by science policy promoted RRI, we were agents of science policy. Therefore, the important reflexive question was, “was science policy objectifying the art science collaboration for its own agenda?” Other disciplinary interests could also be facilitators (for example philosophers, even the artists or natural scientists themselves) by using RRI funding and therefore becoming agents of science policy. By looking at our own role, institutionally we had been able to see “science policy” as the larger facilitator in a general sense, whereas specific disciplinary actors were the facilitators in the specific situation, in our case social scientists. And in the BrisSynBio case, it would later become apparent to me that the performance artists themselves largely did the specific facilitation role even if science policy (funding mechanism) was the general facilitator of an art-science RRI triangle (Fig. 5d).

Because the BBSRC requires their funded biological researchers to commit a percentage of their time to public engagement work, there were attempts to adapt RRI to a public engagement agenda, more so to fulfil BBSRC time requirements than any attempted research laboratory public relations exercise. In the simplest way, most biologists in my field work seem to experience RRI as public engagement. It is but a nuance that to a social scientist RRI also encompasses futures work, reflexivity that locates the researcher and the research project within the bigger picture, and should feedback into the long-term research agenda when future decisions are taken on research direction. The University of Bristol public engagement team seems to have incorporated a more nuanced, plastic definition of public engagement and thus they had the ability to support RRI within BrisSynBio, beginning with the idea that public engagement is two-way communication, and continuing to other nuances like the anticipatory/reflexive aspect of RRI noted in the AREA model. In this case, the use of the term “public engagement” did not have as much of an eclipsing effect on the other qualitative aspects of RRI as I expected to find. But distinguishing inclusive engagement as an aspect of, rather than a synonym for, a fully complete RRI programme (as I argued for in the “Art-Science Exchange” section above) may still be potentially useful.

Public engagement is also the RRI component which forces a research programme to do more than interdisciplinarity; it often requires cross-sectoral interaction, at least in the generic sense that publics are not entirely composed of other academic researchers and thus have different incentive structures and goals. In this, I note that the art-science theoretical framework that I proposed in the above sections is set within an interdisciplinarity framework. This is in contrast to the empirical material I present where the success of the theatre facilitators (largely without direction from policy agents or funders) was in part due to their existing cross-sectoral status relative to the academic researchers. They were a community-based public engagement theatre company, not art theorists or academic researchers, although they did have their own method of experimentation and knowledge criteria. Their relation to BrisSynBio I would position as somewhere between cross sectoral and interdisciplinary (see Fig. 5d above). It is important to remember that in principle RRI must be cross sectoral (as well as interdisciplinary) because it seeks to engage stakeholders, publics with a role in determining scientific research agendas based on societal needs.

## Conclusion

Here I have examined the potential for art-science collaborations to be the basis for deliberative discussions on research agendas and direction. Responsible Research and Innovation (RRI) has become a science policy goal in synthetic biology and several other high-profile areas of scientific research. While art-science collaborations offer the potential to engage both publics and scientists and thus possess the potential to facilitate the desired “mutual responsiveness” (von Schomberg) between researchers, institutional actors, publics and various stakeholders, there are potential challenges in effectively implementing collaborations as well as dangers in potentially instrumentalizing artistic work for science policy or innovation agendas when power differentials in collaborations remain unacknowledged. I have suggested art-science collaborations are processes of exchange which require acknowledgement of and attention to artistic agendas (how can science be a conceptual and material resource for new aesthetics work) as well as identification of and attention to aesthetic dimensions of scientific research (how are aesthetics and affective framings a part of and a specific epistemological resource for scientific research). A common misunderstanding is the expectation that art can be used to promote a “science agenda”. I have therefore suggested the advantage of specifically identifying public engagement/science communication as a distinct aspect of such projects so that aesthetic, scientific or social science/philosophical research agendas are not subsumed to the assumption that the primary or only value of art-science collaborations is as a form of public engagement or science communication to mediate biological sciences research community public messages.

From the evidence of BrisSynBio, there is a potential for art-science collaborations to be a basis for deliberative engagement with publics on research agendas and direction. In some cases, the mode of art makes consideration of difficult science less remote to non-scientific publics and can encourage a more inclusive engagement. They can also encourage reflection by scientific researchers on their own practice and where it is situated within research economies, stakeholder concerns and promissory discourses about the future benefits of synthetic biology.

As part of the practical implementation of such a project, there is usually some facilitation or at least direction from agents responsible for or aware of the RRI policy goals. Thus, I have suggested, it is useful to think of an art-science-RRI triangle. In suggesting this, I encourage a more reflexive research agenda within the Science and Technology Studies (STS) and science communication community—what is the RRI researcher/facilitator role within science policy and the incentive structure that motivates it? In the case presented here, facilitation was in part performed by the artists themselves; developing group processes were part of their specialty. The public engagement office at the university set up the collaboration, some of the RRI theme team participated in the process, and some (apparently quite limited) orientation was given to the theatre group in their initial brief. Nevertheless, what can be considered a useful process emerged. Art-science collaborations can take up many of the questions an RRI process suggests are important. The empirical evidence reviewed here supports the model I propose to guide our framing of art-science within an RRI process but additionally, points to the need to broaden the conception of and possible purposes, or motivations of art. For example, in the case of cross-sectoral collaboration with community engaged art, we can see that the artists already had a clear engagement agenda and experience in developing reflexive processes with a community of non-theatre participants (i.e. community theatre is not the same as academic or high art theatre, nor is it quite the same as commercial theatre) and this experience was applied to the science-theatre project. Thus, whereas the very simplistic guidance model I have proposed here suggested we think of art-science as primarily interdisciplinary, in this case of a community-based theatre, it is necessary to identify also the cross-sectoral aspect of the collaboration. There was an “aesthetics research” interest by the theatre group, but perhaps more interestingly the emphasis of community theatre was more about inclusivity and engagement (their sectoral goal) than aesthetic experimentation with bodily performance (which might have been an academic theatre’s disciplinary research goal), although clearly both were part of their work. Their emphasis was on the process of engaging social relations of a group and theatre (bodily performance) was a means rather than clearly an end in itself. The play was considered a bi-product of the process, intended to be inclusive and reflexive—and in this particular case, intended also to consider futures. Perhaps because of this philosophy of community theatre as engagement, the troupe functioned as the RRI facilitators themselves, in such a way that this initial art-science project was successful as RRI, i.e. encouraged the anticipatory, inclusive and reflexive discussions and provided a model for a research centre that was wrestling with how to understand and enact its RRI requirement. Thus, the centre through this initial process seems to have developed other art-science collaborations and enacted the RRI strand of its obligations in this way.

As one of my BrisSynBio informants put it, “*Invincible* [...] was an immersive site-specific theatre production, that was one of our most important outputs from the whole of BrisSynBio. It was public engagement, it was responsible research and innovation, it was reflexive, it involved a huge number of early career researchers, senior academics, professional practitioners, theatre-makers, and y’know, the success of that endeavour unlocked a whole load of ‘kind-of’ strategic thinking about what RRI is, how we can do RRI at Bristol, what the benefits are for all of the parties involved, and I think that’s really the point at which we realized the power of the arts in unlocking some of those questions for us”.

## Footnotes

1 The BrisSynBio website is available at http://www.bristol.ac.uk/brissynbio/ (last accessed Nov 23, 2019). See https://bbsrc.ukri.org/research/programmes-networks/synthetic-biology-growth-programme/ (last accessed Nov 23, 2019). Although formal funding of the centre finished in June 2019, the University of Bristol maintains an ongoing programme in Biodesign which succeeds and was setup by BrisSynBio. See http://www.bristol.ac.uk/research/institutes/biodesign/ (last accessed Nov 23, 2019).

2 See also Rosenberg [5], Arthur [6] and David [7].

3 https://ec.europa.eu/programmes/horizon2020/en/h2020-section/responsible-research-innovation (last accessed Dec 1, 2019)

4 See https://epsrc.ukri.org/research/framework/area/ (last accessed April 4, 2019). The UK Biotechnology and Biological Sciences Research Council (BBSRC) does not have specific guidelines but encourages social responsibility in science.

5 Bimber [10], Schot and Rip [11], Fisher E [12], Guston and Sarewitz [13], Fisher et al. [14], Fisher E, Guston DH [15], Barben et al. [16], Guston [17, 18], von Schomberg [9], Wynne [19], Owen et al. [20], Stilgoe et al. [21]. Loosely based on Stilgoe et al. [21], the Environment and Physical Sciences Research Council in the UK suggests an AREA model for ensuring RRI: to Anticipate, Reflect, Engage, Act. See note 4 above.

6 At the hubristic end of the scientific imagination, this reorganization/appropriation is sometimes labelled as the creation of life. The patterns that are being emulated/rearranged are all preformulated during millions of years of evolution.

7 BrisSynBio maintains a number of start-ups and promotes mixed path roles linking the academic and entrepreneurial.

8 For the original reference, see Mackenzie [42].

9 For this reason, when trying to satisfy a cost-benefit arithmetic for both parties, public engagement may be a legitimate deliverable in an art-science exchange. My argument is not that public engagement is itself a bad thing, far from it, but rather, that public engagement should not be a substitute for a more reflexive self-understanding of science as a discipline with many disciplines within itself (likewise art), an understanding perhaps facilitated by a successful art-science collaboration.

10 http://www.bristol.ac.uk/brissynbio/about-brissynbio/ethics-and-responsible-research-and-innovation/https://www.youtube.com/watch?v=rk25Fl4ajEY (last accessed Dec 1, 2019)

11 Most of my empirical material regards cross-sectoral engagement with stakeholders exploring attitudes to gene editing and wheat breeding.

12 https://www.synenergene.eu/ (last accessed Dec 1, 2019)

13 http://www.perform-research.eu/ (last accessed Dec 1, 2019)

14 https://www.youtube.com/watch?v=71K6h3wg1i8 (last accessed Dec 1, 2019)

15 http://www.kiltertheatre.org/projects_workshops/futures (last accessed Dec 1, 2019)

16 The reason this question was important for the framework I had developed is discussed in the “Discussion of Findings” section below, particularly in the discussion of Fig. 5.

17 http://www.perform-research.eu/ (last accessed Dec 1, 2019)

18 A BBSRC/EPSRC funded Centre for Doctoral Training (CDT) in Synthetic Biology was inclusive of BrisSynBio PhD students and also two other universities. http://www.synbio-cdt.ac.uk/ (last accessed Dec 1, 2019)

19 “General Certificate of Secondary Education”; In the UK, these exams precede two years of “A level” studies before applying to a university.

20 In principle philosophers, historians or other disciplines could also have been the facilitators. Most art science collaboration takes place without facilitators other than the artists/scientists themselves.
